# Anomaly detection in railway bridges using imaging techniques

**DOI:** 10.1038/s41598-023-30683-z

**Published:** 2023-03-08

**Authors:** Paolo Russo, Marco Schaerf

**Affiliations:** Department of Computer, Control, and Management Engineering “Antonio Ruberti”, Sapienza University of Rome, Via Ariosto 25, 00185 Rome, Italy

**Keywords:** Civil engineering, Computer science

## Abstract

The monitoring of the structural health of infrastructures is a very important topic in structural engineering, but unfortunately, there are few established techniques that are applicable in a wide range of situations. In this paper, we present a new method that adapts image analysis tools and methodologies, taken from the field of computer vision, and applies them to the monitoring signals of a railway bridge. We show that our method correctly identifies changes in the structural health of the bridge with very high precision, thus providing a better, simpler, and more general alternative to current methodologies used in the field.

## Introduction

In this paper we show how the use of imaging and machine learning techniques can help monitor a physical structure’s response during its lifetime. After the seminal work of Rytter^1^ and the interesting analysis of Cantero et al.^2^, many other authors have proposed methodologies to address this problem, here we try to improve on the analysis performed on the data collected by Maes and Lombaert^3^ and analyzed by the same authors in^4,5^. This dataset contains the data series of the sensors installed on the railway bridge KW51 (located near Leuven, Belgium) collected before, during, and after works that modified the bridge to resolve a construction error.

We propose *Imaging techniques for Anomaly DEtection* (IADE), a solution which relies on first applying the Synchrosqueezing Continuous Wavelet Transform (Synchrosqueezing CWT introduced by Daubechies and Maes^6^ and then further explored in^7,8^) to the signals produced by the sensors, and then transforming the output into an image by applying a colormap function to the frequency-domain and time-domain wavelets. Each time series of accelerometers is thus transformed into an RGB image and then sent to a pre-trained deep network called DenseNet-161, introduced by Huang et al.^9^ The outputs of the network are then collected and fed to a network composed of 2 fully connected layers with a final layer outputting a binary classification (normal bridge/modified bridge).

We show that our IADE  framework has high accuracy on the proposed dataset, with results similar to those obtained with classical methods of structural engineering while being far more general and easier to implement.

## Methods and tools

**Figure 1:**
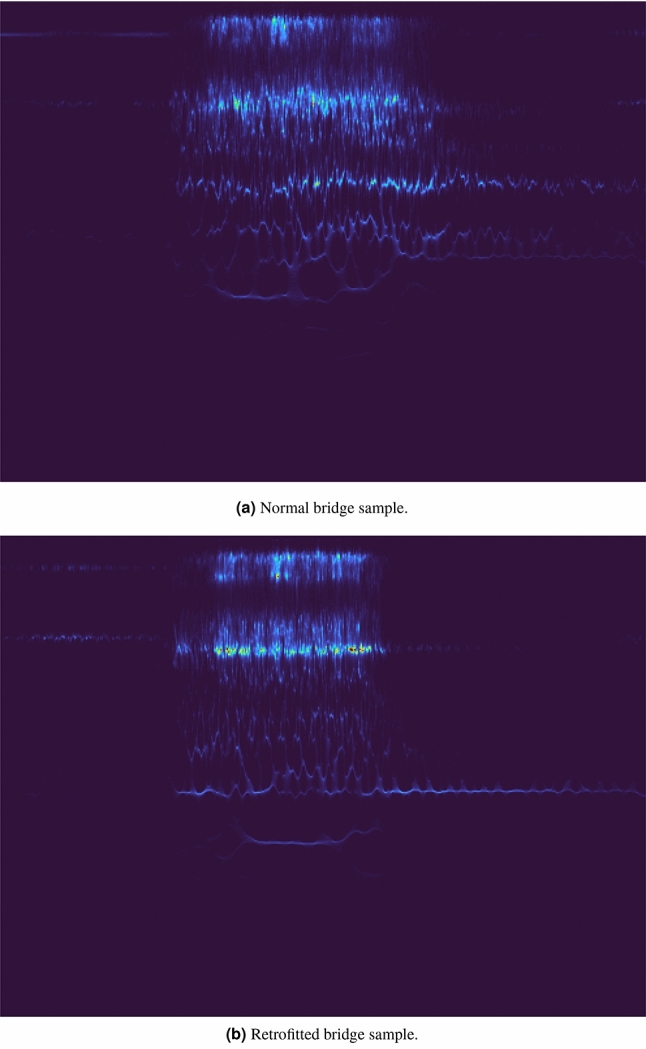
Example of images produced by applying the SCWT function on two samples, followed by the application of *turbo* colormap function. (**a**) Image refers to a normal bridge sample, while (**b**) refers to a retrofitted bridge sample.

The goal of this work is to show that machine learning and imaging techniques can be successfully applied to classification problems in apparently unrelated fields such as physical structures monitoring even when the monitoring sensors do not provide any visual output. We build IADE on the data and methodologies used by Maes and Lombaert. More precisely, the dataset containing the data series produced by the sensors (mostly accelerometers) installed on the KW51 railway bridge near Leuven, and collected in a 15 months period of time during which the bridge has been modified to compensate for a construction error^3^.

The analysis of the collected data, performed by the same authors^4,5^, shows that even experts in the field of vibration monitoring struggle with recognizing whether the data comes from the original bridge or the modified one. Moreover, the analysis performed in^5^ is quite specific to the KW51 bridge, thus not leading to a general methodology that can be applied in a number of different situations.

With the proposed methodology IADE we investigate the use of techniques developed in the machine learning and image processing communities in order to test their applicability in this specific field of research.

More in detail, in order to tackle the task of anomaly detection on railway bridges, we cast the problem into a binary classification task and we propose the following two-steps approach to solve it:

- Transformation of the accelerometer data corresponding to the train passage with the Synchrosqueezing Continuous Wavelet Transform algorithm, followed by a colormap function to obtain an RGB image representation.
- Deep neural network anomaly prediction by accumulating visual features corresponding to each image.

In the next subsections, we will describe in detail the above steps, while the results obtained by IADE will be shown in section “Results”.

### Synchrosqueezing continuous wavelet transform

In order to transform the accelerometer time series into images, we first apply to the signals the Synchrosqueezing Continuous Wavelet Transform (SCWT) introduced by Daubechies and Maes^6^. This technique is based on a Time-frequency (TF) representation of the signal and has been further refined in^7,8^. As discussed in^7^, this formalism allows for the representation of many signals common in the engineering field that have several components, all reasonably well localized in TF space, at different locations. These so-called “non-stationary” signals are difficult to characterize using other similar representations such as linear or quadratic ones. In recent years, the Continuous Wavelet Transform has been used in several studies on bridge damage detection, including^2,10–12^ where various forms of Continuous Wavelet Transforms have been successfully applied to the analysis of sensor signals applied to bridges. In all of these studies, SCWT and other similar transforms have been repeatedly shown to be a very effective way to extract information from sensor data in this specific field of research. Moreover, the SCWT can be considered a general-purpose transformation with respect to the input signal, requiring very loose assumptions on the signal characteristics^7^, thus avoiding the risk of overfitting on specific anomaly signals.

The output of the Synchrosqueezing Continuous Wavelet Transform, in particular, the frequency-domain and the time-domain wavelets, contains a lot of useful information on the analyzed data, but it can be difficult, even for a human expert, to figure out whether it shows an anomalous behavior or not. Moreover, the domain transformation from 1D signals to 2D temporal-frequency data enables the production of an RGB image by applying a simple color mapping procedure; this enables the exploitation of powerful, pre-trained deep neural networks on the resulting images for accurate and robust anomaly detection.

### 2D transformation and color mapping

The Synchrosqueezing Continuous Wavelet Transform briefly described in the previous paragraph produces as output a frequency-domain wavelet and a time-domain wavelet, which can be mapped into a 2D plane by putting the former on the y-axis and the latter on the x-axis. After a normalization step, this matrix can be interpreted as a grayscale image with a single color channel. However, in order to exploit the power of deep neural network features pre-trained on the ImageNet dataset, we have mapped this grayscale image to an RGB, 3-channel image which matches the required 3-dimensional input of pre-trained on ImageNet deep neural networks. We preferred to avoid adopting one of the most used colormap functions, that is the *Jet* function, which is widely used but has been shown not to be perceptually uniform, with clearly distinct “bands” of color around the regions of the yellow and cyan color. Instead, we opted for the *turbo* colormap function developed by Google which provides smoother transitions between color channels. More details on turbo and jet colormaps can be found on the Google AI blog (https://ai.googleblog.com/2019/08/turbo-improved-rainbow-colormap-for.html). However, we would like to remark that IADE pipeline is agnostic with respect to the chosen colormap, which produces little to negligible impact on the overall performances. As Synchrosqueezing Continuous Wavelet Transform library we used the *ssqueezepy* one provided by Muradeli^13^ which is a Python implementation of the MATLAB Synchrosqueezing Toolbox developed by Thakur et al.^8^ An example of the SCWT processed by the colormap function can be seen in Fig. 1, where two samples are shown, belonging to the normal bridge and the retrofitted bridge respectively. We would like to remark that, although the output of SCWT is well defined as it represents the instantaneous frequency content at any given time, a physical interpretation of the actual SCWT output is far from being trivial and is still an open problem in the signal processing field^14,15^. Luckily, it is not required to explicitly characterize the actual image features as IADE  is able to automatically learn the best visual features which correspond to 1D signal characteristics in both normal and retrofitted bridges. The time-frequency signal content of the normal and retrofitted bridges are different and our model is able to learn those differences and perform the classification with high accuracy, as reported in section “Results”.

### Deep features accumulation

**Figure 2:**
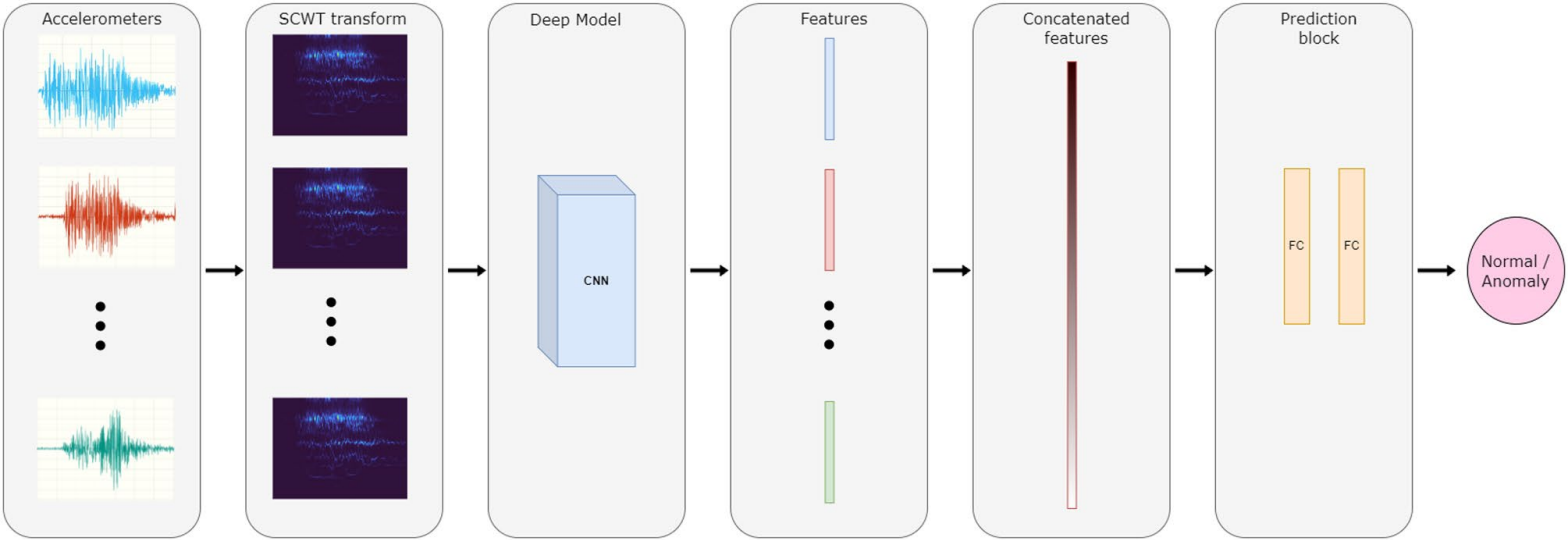
IADE  proposed pipeline. The first block represents the raw input signals coming from the accelerometers, while the second block is showing the RGB image obtained by transforming the input with the SCWT function and *turbo* colormap. The remaining blocks depict the deep neural network module with the features accumulation step and the final binary classification.

The application of the previous step to the accelerometer data produces as output a series of images that contain an encoding of the temporal-frequencies information. In principle, each of those images can be used to perform a binary classification with the use of a deep neural network. Moreover, the most powerful available deep models accept as input a single RGB image, thus restricting the use of multiple images at the same time. With IADE  we propose a simple fusion schema to perform anomaly detection with a deep neural network by exploiting all the accelerometer images at the same time: for each image, the activation maps produced at the latest neural network convolutional layer are extracted; the activation maps obtained are then accumulated in a single tensor by performing concatenation along the feature dimension. The resulting tensor is fed to two fully connected layers which perform feature reduction and binary classification respectively. The whole IADE  pipeline is represented in Fig. 2.

The IADE  pipeline can in principle be applied to any deep convolutional neural network; in the next section, we show the results obtained by using DenseNet 161^9^ model as the backbone network, as it provides good pre-trained visual features and manageable training complexity.

During the training phase, the backbone network has been fine-tuned on the training data by initializing the weights with the ImageNet pre-trained weights, and by applying a small learning rate ($10^{-4}$ with Adam optimizer) to the weights training. The two fully connected layers have been initialized from scratch with a standard Xavier initialization and have been jointly trained with the backbone network at the same learning rate.

The advantages of IADE  approach can be summarized as follows:

- it enables the exploitation of deep neural networks by converting each 1D accelerometer signal to an RGB 2D image by applying the SCWT transformation and a standard color mapping.
- it makes use of several accelerometer data at once, to perform deep anomaly detection in a multivariate fashion; the resulting binary classification is more robust to single accelerometer outliers, thus increasing the overall accuracy.
- The use of features accumulation enables the exploitation of pre-trained deep neural networks, encouraging the re-use of useful visual features and at the same time avoiding the employment of an ensemble of networks (one for each accelerometer) which would otherwise produce a cumbersome model, more prone to overfitting.

For this reason, we decided to feed the graphical outputs of the Synchrosqueezing Continuous Wavelet Transform of our time series to a (predefined and pre-trained) image analysis network in order to extract a feature vector for each time series. We choose the DenseNet 161 network, belonging to the family of DenseNets introduced by Huang et al.^9^, because of its properties and performances on the ImageNet dataset^16^.

The feature vectors obtained are concatenated and provided as input to a network composed of a sequence of 2 fully connected layers, where the last one performs a binary classification on Normal bridge/Abnormal bridge categories.

## Dataset

In this section, we describe the railway bridge KW51 and the related data. The following quote is a summary of the properties of the bridge taken from the work of Maes and Lombaert^4^:

The subject of this study is a steel railway bridge in Leuven, Belgium, referred to as railway bridge KW51. The bridge is of the bowstring type and has a length of 115 m and a width of 12.4 m. A maximum speed of 160 km/h is imposed. The bridge is used by passenger trains and was opened for traffic in 2003.

The railway bridge has been monitored since October 2, 2018. In the period from May 15 to September 27, 2019, the bridge was retrofitted to resolve a construction error that was noticed during the inspection. The retrofitting consisted of strengthening the connections of the diagonals to the arches and the bridge deck. The data collected by Maes and Lombaert^3^ concern a period of 7.5 months before the retrofitting (October 2, 2018–May 15, 2019), the period of the retrofitting (May 15–September 27, 2019), and a period of 3.5 months after the retrofitting (September 27, 2019–January 15, 2020).

The railway bridge is instrumented with the installation of accelerometers on the bridge deck, strain gauges on the bridge deck and the diagonals connecting the bridge deck to the arches, strain gauges on the rails, a thermocouple below the bridge deck, and a relative humidity (RH) sensor. The system was extended with accelerometers on the arches in September 2019 (stage 2) and with displacement sensors at the bearings of the bridge in October 2019 (stage 3).

Unfortunately, some sensors have been kept off during some periods of the evaluation study (see Fig. 11 in Maes and Lombaert^4^ paper); in order to build a consistent dataset, we selected the accelerometers named *aBD11Az, aBD17Ay,aBD17Az, aBD17Cx, aBD23Ay, aBD23Az* which have fully acquired data starting from 1st of November 2018 to 1st of January 2020. During that period, a total of 813 train passages have been collected. We divided the data into training and testing splits by assigning to the testing set the train passages data collected during November 2018 (normal bridge), April 2019 (normal bridge), June 2019 (retrofitted bridge), and December 2019 (retrofitted bridge), while assigning the other data to the training set. This ensures that both training and testing sets have train passages at different weather temperatures, which helps to avoid any temperature bias in the dataset which could affect the model generalization capabilities. The described dataset contains 591 training samples and 222 testing samples; each sample is constituted by six accelerometer signals converted to six corresponding RGB images by the Synchrosqueezing Continuous Wavelet Transform function and a simple colormap described in section “Synchrosqueezing continuous wavelet transform”. The images are resized to 224 $\times$ 224 resolution to match the input size required by the deep model; no crop function has been applied to avoid the loss of important time-frequency information.

## Implementation details

The Synchrosqueezing Continuous Wavelet Transform has been applied with the *mu* and *nv* values set to 4.5 and 32 respectively. However, due to the robustness of the aforementioned transform, other different values can be used with similar performances. Among the several possible choices ( *Generalized Morse Wavelets*, *Bump*, etc) we used the *morlet* wavelet function. The RGB images produced by the colormap step have been normalized with zero mean and unit variance calculated over the training set before being fed to the deep neural network. The two fully connected layers are composed of 1024 neurons each and interspersed with ReLU non-linearities, with a dropout value of 0.5 applied on the output of the first connected layer, which is receiving as input 13,248 (2208 $\times$ 6) aggregated visual features. The training has been performed over 20 epochs using the Adam solver, and the reported accuracy is the mean value over 10 runs. Experiments conducted with the use of standard augmentation functions (random cropping, mirroring, zooming, added pixel noise) showed that the best performances are obtained without any data augmentation function at all. We used the standard categorical, cross-entropy loss function in order to train the network, with a Softmax layer, following the last fully connected layer, which produces the model prediction. IADE  has been implemented in Python with the use of PyTorch version 1.13 (https://pytorch.org) and the trainings have been performed on an Nvidia TitanX with 12 GB of GPU memory. The training batch size has been set to 16. As the required GPU memory scales up with the number of sensor images fed as input, it is advised to use the Pytorch gradient accumulation feature in order to avoid reducing the batch size value, which could eventually bring training instabilities.

## Results

In this section we detail the results we obtain in the binary classification of normal and retrofitted KW51 bridge using IADE, described in section “Methods and tools”. The metrics used to assess IADE  performances are *Accuracy*, *Precision* and *Recall*, defined below:

1 $$\begin{aligned} Accuracy= \frac{TP+TN}{TP+TN+FP+FN}\quad Precision= \frac{TP}{TP+FP}\quad Recall= \frac{TP}{TP+FN}, \end{aligned}$$

where TP, TN, FP, and FN stand for *True Positive*, *True Negative*, *False Positive*, *False Negative* respectively. Accuracy is a metric that represents how the model performs across all task categories, as it is calculated as the ratio between the number of correct predictions to the total number of predictions. Precision instead is a metric that measures the proportion of positive predictions that are actually correct. Finally, Recall shows the proportion of actual positive data points that are correctly classified as positive by the model (true positive), out of all the actual positive data points. Working on anomaly detection on bridges, we associate the normal bridge sample with the negative class and the retrofitted bridge sample with the positive (or anomaly) class. Consequently, in our task, we define as true positive the number of anomaly bridge samples that are correctly classified as an anomaly, while the normal bridge samples misclassified as an anomaly are the false positive. In the same way, we define true negative as the number of normal bridge samples which are correctly classified as normal, and finally the false negative represents the number of anomaly bridge samples misclassified as normal.

Given these definitions, Accuracy shows a general representation of the model performances among both normal and retrofitted (anomaly) categories, while Recall measures IADE  capacity to correctly identify all anomalous situations within our dataset and Precision focuses on the correctness of the total amount of anomaly predictions.

Table 1 summarizes the Accuracy, Precision, and Recall obtained by IADE  when getting the full input (accelerometer 1-6) with respect to the same method but on a single accelerometer data. The high accuracy obtained by IADE  when working on six signals as inputs clearly shows the soundness of our approach, with an accuracy value greater than 97%. At the same time, the lower accuracies of the models working on single input show the importance of deep features accumulation. In fact, the best accuracy obtained by the model working on signal 2 (which we conjecture corresponds to the accelerometer near the upgraded bridge part) still lags behind the full model, with more than a 5% of difference in accuracy. Moreover, our proposed method is agnostic concerning which accelerometer carries the most informative signal, thanks to its multivariate approach.

The high value of Precision obtained in both single and accumulated features means that a high number of predicted positive samples are genuinely positive, or correspondingly that the number of false negatives (samples belonging to retrofitted bridge miss-classified as a normal bridge) is low. On the contrary, the Recall values measured in our experiments are generally lower than Precision ones, which means that a proportion of actual positive samples are incorrectly classified as negative. However, IADE  working on accumulated features exhibits a Recall value only 1.8% lower than the Precision value, thus sensibly improving the Recall performances with respect to a single signal as input. This analysis is confirmed by the values of per-class accuracy: IADE  applied on six accelerometers produces 98.1% accuracy concerning the normal bridge category, and 96.3% accuracy concerning the retrofitted bridge category, which shows again that the classification of retrofitted (positive class) bridge samples is slightly harder than the other class. As an additional experiment, we performed a model training starting from random network weights instead of relying on the usual, pre-trained on ImageNet weights, to assess the importance of pre-training weights even when the target task is sensibly different with respect to the original ImageNet task. When trained from scratch the model has shown a maximum accuracy value equal to $95.1\%$, with a difference with respect to the pre-trained case of 2.2 points. Moreover, the training phase exhibited a strong instability, with the accuracy values wildly oscillating up to 30 points, which are non-existent when working with a pre-trained model. This peculiar behavior, together with the performance hit, suggests that the use of a pre-trained model is crucial even in sensibly different contexts, as is the task of anomaly detection applied to SCWT transformed data.

**Table 1:** IADE  Results of our method, with six accelerometer images as input, with respect to the results from the single accelerometer data as input. Precision and recall use the retrofitted bridge category as the positive class and the normal bridge category as the negative class.

Method	Accuracy (%)	Precision (%)	Recall (%)
IADE (signals 1–6)	**97.3**	**98.2**	**96.4**
IADE (signal 1)	90.1	94.1	85.6
IADE (signal 2)	91.9	96.0	87.4
IADE (signal 3)	89.1	92.2	85.6
IADE ( signal 4)	87.8	90.4	84.7
IADE (signal 5)	87.3	94.6	79.3
IADE (signal 6)	89.2	93.1	84.7

Best results are reported in bold.

## Discussion and conclusions

In this paper, we have presented the results of our analysis of the signals captured by the sensors installed on the KW51 bridge during the transit of the trains before and after the modification of the bridge.

The proposed IADE  consists of transforming the sensor signals via the SCWT transformation and its encoding in images. This methodology has been applied to a situation where the signals available before and after the retrofitting were only 6, thus making the overall architecture more manageable. We believe that our architecture can efficiently work with up to 20 signals, producing a high accuracy and avoiding the need to select the most informative signals. The use of IADE  reduces the need for human experts performing anomaly detection with classical structural engineering methods. Nevertheless, our method can be exploited in parallel to the prediction of a human expert to get a confirmation and thus increase the prediction robustness.

Moreover, we believe that our methodology can be applied to many other situations since it can be easily extended to more complex cases along the following lines:

- If the transformed signal is not adequate for the classification task, many alternative transformations can be used without affecting the overall pipeline.
- If the number of relevant signals increases, we can apply dimensionality-reduction techniques such as Principal Component Analysis (PCA)^17^ or others so to reduce the number of relevant signals.
- if the number of features becomes too high, it is possible to use image processing networks that produce a more limited number of features per image (using for example SqueezeNet^18^ that produces fewer features per image).

Regarding the choice of the transformation function, the obtained results demonstrate the power of SCWT to capture salient signal characteristics. The SCWT function is an agnostic transformation function with respect to the input signal characteristics, having very loose requirements on which type of signal is suitable for such transformation. However, it is not possible to rule out that other transformation functions could be even better suited for a specific anomaly detection task. In this sense, IADE  is ready to work seamlessly with any kind of 1D$\Rightarrow$2D transformation, thanks to its ability to develop ad-hoc visual features.

Future works could also focus on adding auxiliary information to the model to strengthen the classification—when available, additional data such as train weight, speed, and length could be used by the model as an extra feature and thus making the classification phase more robust. Finally, our method could be extended for bridge damage localization with a second network branch and a multi-task learning approach.

## Data Availability

The dataset exploited to assess the performance of the proposed method has been collected by Maes and Lombaert^4^ and has been made publicly available for download by the authors at the following link: https://zenodo.org/record/3745914. The dataset is released by the authors under the copyright licence *Creative Commons Attribution Non Commercial Share Alike 4.0 International*https://creativecommons.org/licenses/by-nc-sa/4.0/legalcode.
